# The impact of rainfall and school break time policies on physical activity in 9-10 year old British children: a repeated measures study

**DOI:** 10.1186/1479-5868-8-47

**Published:** 2011-05-24

**Authors:** Flo Harrison, Andrew P Jones, Graham Bentham, Esther MF van Sluijs, Aedín Cassidy, Simon J Griffin

**Affiliations:** 1 School of Environmental Sciences, University of East Anglia, Norwich, NR4 7JT, UK; 2 Medical Research Council Epidemiology Unit, Institute of Metabolic Science, Box 285, Addenbrookes Hospital, Hills Road, Cambridge, CB2 0QQ, UK; 3 School of Medicine, University of East Anglia, Norwich, NR4 7JT, UK

## Abstract

**Background:**

The weather may be a driver of seasonal patterns in children's physical activity (PA). A better understanding of the relationships between weather and PA may help increase children's PA. This study aims to examine the association between PA and rainfall in 9-10 year old children, and how it may be modified by school policies.

**Methods:**

1794 participants in the SPEEDY study in Norfolk, UK recorded PA using ActiGraph accelerometers over up to six days in the summer term of 2007. Multilevel regression models were used to determine the day-by-day association between rainfall and minutes spent sedentary, in moderate-to-vigorous PA (MVPA), and average counts per minute (cpm) over the whole day (07:00-21:00) and the lunchtime period (12:00-14:00). School policies for break times in bad weather were fitted as interaction terms with rainfall.

**Results:**

Relative to days with no rain, children spent 9.4 minutes (95%CI 7.0 to 11.9) fewer in MVPA, were sedentary for 13.6 minutes (8.8 to 18.4) more, and accumulated 85.9 cpm (66.2 to 105.5) fewer over the whole day on the wettest days. Children allowed to play outside in wet weather showed the lowest lunchtime PA levels on the wettest days, undertaking 9.8 minutes (6.2 to 13.5) fewer MVPA, 16.1 minutes (10.3 to 21.9) more sedentary, and accumulating 408.0 cpm (250.9 to 565.1) fewer than those allowed to be active indoors.

**Conclusions:**

Rainfall is negatively associated with PA in primary school children, but providing indoor physical activities in wet weather may help children maintain physical activity levels irrespective of rainfall.

## Background

Regular physical activity has been shown to benefit the health of children [1,2] and adults [3] and there is evidence that physical activity habits track from childhood into adulthood [4]. An understanding of the factors that encourage or inhibit children's participation in physical activity is therefore important in promoting healthy behaviours throughout the life course.

Physical activity in children exhibits seasonal patterns [5-7] with levels typically higher in spring and summer relative to winter months. Climatic factors have been proposed as potential drivers of these trends [8], and recent reviews reflect the growing interest in the effect that weather (including rainfall, temperature, and sunshine) has on activity levels [9]. In their review of the correlates of physical activity in children, Sallis et al [10] noted that time spent outdoors was consistently associated with higher levels of physical activity, leading to the possibility that weather which inhibits the potential for outdoor play may lead to lower activity levels. Despite this, few studies have looked at the associations between physical activity and weather in children. Of those that have, Bélanger et al [11] found a 2-4% reduction in the number of physical activity sessions (derived from a seven day recall) undertaken per day for every 10mm increase in rainfall while Duncan et al [12] reported a 8-11% decrease in weekday pedometer-recorded step counts amongst children during moderate (1.1-4.9mm) rainfall relative to days with no rain. Children spend a large amount of time at school, and physical activity at school break time can make a significant contribution towards overall activity levels [13], but it is not known how school policies may impact the relationship between weather and physical activity.

Using a well characterised sample of children living and studying in Norfolk, England, this study aims to identify associations between objectively measured physical activity outcomes on school days and rainfall in 9-10 year old children. It also examines how these associations may be modified by school policies towards outdoor play during break times.

## Methods

### Recruitment

The SPEEDY study (Sport, Physical activity and Eating behaviour: Environmental Determinants in Young people) was set up to investigate individual and collective correlates of diet and physical activity behaviour of Year 5 (aged 9-10) children across the county of Norfolk, UK. Ethical approval for SPEEDY was obtained from the University of East Anglia local research ethics committee. The study's methods are described in detail elsewhere [14] and so are only briefly recounted here.

Schools across Norfolk with at least 12 Year 5 pupils were sampled according to a stratification by urban/rural status [15]. Ninety-two schools took part in the study, and 2064 children were recruited into SPEEDY; 57% of the 3619 invited to participate. Data collection was performed during the summer term (April to July) of 2007. Teams of trained Research Assistants performed measurements at participating schools according to standard operating procedures and fitted children with an accelerometer, which was collected at the schools 8 days after the measurement day.

### Physical Activity

ActiGraph accelerometers (GT1M, Actigraph LCC, Pensacola, US) set to measure at 5-second intervals, were used to assess free-living activity over seven days. The children wore the accelerometers during waking hours on the right hip. The first day of data collection was removed from all files as there is evidence that children are more active than normal during this time [16] and 10 minutes of continuous zero counts were classified as 'non-wear time'. 'Wear time' was derived by subtracting minutes of 'non-wear time' from the total minutes in a given period. For the analyses presented in this paper, only days where the children attended school were included. We also excluded days for which there were fewer than 500 minutes of valid data. This period was chosen as being appropriate for children in the 9-10 year old age group. Physical activity during two time periods were investigated; the whole day (7am to 9pm) and the lunchtime period (12pm to 2pm). For each of these periods, we derived three outcome variables: the number of minutes children spent in sedentary (≤100 counts/minute) activity, activity of moderate to vigorous physical activity (>2000 counts/min; MVPA), and mean counts per minute (cpm), a measure of the average intensity of physical activity. The chosen threshold for MVPA equates to a walking pace of roughly 3 km/h in children [17] and has previously been used in the study of physical activity and adiposity in this age group [18].

### Rainfall

As temperatures in Norfolk were relatively benign during this specific period (daily maxima ranged between 10.5 and 23.5°C), rainfall was used as an indicator of how appropriate the weather conditions may be for outdoor play. Rainfall data were derived from the UK Meteorological Office's MIDAS Land Surface Observation Stations Data [19]. Two stations in Norfolk were identified as having near continuous hourly data for the whole study period: Marham, in the southwest of the county, and Weybourne on the north coast (50km apart). For these stations we extracted hourly rainfall (mm), and summed values to provide daily (7am to 9pm) totals. Rainfall at the two stations were highly correlated (r = 0.876, p < 0.001), so when data were available from both we calculated the mean value (data from both stations were available on 60 days; 92% of the study period), otherwise data from one station was used. There were no days for which data were unavailable at both stations.

### School policies

The head teacher at each of the participating schools was asked to complete a questionnaire, designed especially for this study, which included questions on physical activity and food related facilities, policies and learning opportunities. This included two questions on the school's policies relating to break times in bad weather. Head teachers were asked to choose from a list of policies which best described their rules relating to where children can play during breaks (including lunchtime). The policies were:

a. It is compulsory for all children to play outside, irrespective of the weather.

b. When the weather allows, it is compulsory for all children to play outside. However, all children are kept inside in bad weather.

c. When the weather allows, it is compulsory for all children to play outside. However, if the weather is bad, they are allowed to play inside or outside.

d. The children are allowed to play both inside and outside, irrespective of the weather.

e. It is compulsory for all children to play inside, irrespective of the weather.

Furthermore they were asked whether children were allowed to do a range of activities (use a computer, watch TV or videos, use the school's sports equipment, play a ball game indoors, play a running game indoors, play ball games outdoors) either 'always', 'in bad weather' or 'never'.

We grouped together schools who provided indoor physical activities (indoor running games or indoor ball games) in bad weather regardless of their answer to the question about where children were allowed to play. Remaining schools (who did not allow indoor physical activities) were then categorised as either those who allowed pupils outdoors in wet weather (answering a, c, or d, to the question about where children were allowed to play), or those where children were kept indoors in wet weather, resulting in three policy groups. ('Indoor PA allowed', 'No indoor PA allowed', 'Outdoor PA allowed').

### Covariates

Research assistants collected anthropometric data according to standard operating procedures, including foot-to-foot bioelectrical impedance using a Tanita scale (type TBF-300A). Fat Mass Index (FMI = FM(kg)/height(m)2) was calculated using previously validated equations [20]. In the school questionnaire, head teachers were asked to indicate how long break times lasted, whether the school had access to a specific indoor hall for gym or sports, and in which area children could play outdoors on a fine day in the summer. The proportion of pupils at each school eligible for free school meals was obtained from the local education authority and the Index of Multiple Deprivation (IMD) score [21] for the school neighbourhood was extracted based on the lower super output area the school fell in. The IMD score is a composite index comprising measures of income, employment, health and disability, education, skills and training, barriers to housing and services, living environment and crime.

### Statistical analysis

All statistical analyses were performed in 2010 using Stata IC version 11. To take account of potential non-independence of outcomes (each child provided more than one day of data, and groups of children attended the same schools) multi-level regression models were used, with day at level 1, child level 2, and school level 3. Rainfall was banded into four categories, days of no rainfall (44.4% of days in the study period) formed the bottom category, with remaining days banded into tertiles, and analysed as a categorical variable with a test for trend. First, the association between rainfall and each of the accelerometry outcome variables in each time period was assessed in models adjusted for sex, log-transformed FMI, length of break time at school, and minutes of wear time (in the sedentary and MVPA models). Second, we included an interaction term between rainfall and break time policies in each of the models. The estimates from these were used to calculate the physical activity outcome (and associated standard errors) in each rain category/policy combination, holding the covariates at mean values.

## Results

Of the 2064 children recruited in the SPEEDY study, 1866 (90.4%) recorded at least 500 minutes of physical activity data on at least one week day. A further 70 were excluded as 12 did not provide valid impedance data, 31 were measured at a pilot school in February, and 27 only provided data during the half-term break. This resulted in a final sample of 6334 measurement days for 1794 (86.9% of original sample) participants at 90 schools. There was no difference between those included and excluded in terms of age, sex and FMI (all p > 0.05).

Summaries of the characteristics of the pupils included in these analyses are shown in Tables Table 1. Slightly more girls (55%) than boys were included. Girls generally had higher FMI, and were less active than boys; they spent fewer minutes in MVPA, more minutes sedentary, had lower average counts per minute, both over the whole day and during the lunchtime period (all p < 0.05).

Table 2 summarises the school characteristics of the schools in the analysis. Seven schools provided options for indoor physical activities in wet weather. Of the remaining 83 schools, 11 reported allowing their pupils outdoors in wet weather, but of these only three did not also provide the option for indoor play. The schools which allowed indoor physical activity in wet weather were more likely to have a separate indoor sports hall, had a greater area of outdoor play space per child, a greater proportion of pupils eligible for free school meals, and were more likely to be in a socioeconomically deprived area. However, as the number of schools in this policy group was small, these differences in prevalence were not statistically significant (p>0.05).

**Table 1:** Characteristic of study participants

	**Mean (standard deviation)**		
	**All**	**Girls**	**Boys**
			
Number of participants	1794	986	808
Age (years)	10.25 (0.30)	10.26 (0.30)	10.25 (0.31)
Fat Mass Index (Fat Mass(kg)/height(m)^2^) *	5.82 (2.60)	6.40 (2.65)	5.10 (2.36)
Days for which PA data recorded	3.53 (0.82)	3.55 (0.80)	3.51 (0.85)
Whole day (07:00- - 21:00)...			
Minutes sedentary *	466.1 (63.5)	474.9 (61.7)	455.3 (64.0)
Minutes MVPA *	72.6 (29.3)	64.46 (25.1)	82.8 (30.9)
Counts per minute *	618.6 (244.5)	575.0 (235.3)	672.4 (245.0)
Lunchtime (12:00 - 14:00)...			
Minutes sedentary *	71.6 (12.9)	74.8 (11.9)	67.6 (12.9)
Minutes MVPA *	14.7 (7.8)	12.1 (6.1)	17.8 (8.4)
Counts per minute *	712.8 (325.9)	619 (273.2)	828.3 (347.9)

* difference between girls and boys significant p < 0.05

**Table 2:** Summary of the characteristics of schools in difference break time policy groups.

	**Number (%) or mean (SD)**			
	
	**Indoor PA allowed**	**No indoor PA allowed**	**Allowed outside**	**All**
	
Number of schools	7 (7.8%)	72 (80.0%)	11 (12.2%)	90
Number of participants	134 (7.5%)	1484 (82.7%)	176 (9.8%)	1794
Number of days	443 (7.0%)	5249 (82.9%)	642 (10.1%)	6334
Length of lunch break (minutes)	60.0 (0.0)	58.3 (6.5)	54.5 (11.1)	57.9 (7.0)
Number of pupils in year group^a^	46.4 (26.7)	38.9 (26)	32.8 (27.2)	38.8 (26.5)
School has access to an separate gym^b^	6 (85.7%)	44 (61.1%)	6 (54.6%)	56 (62.2%)
Playground area (m^2 ^per child)^c^	74.2 (61)	60.1 (42.7)	58.6 (51.3)	61.0 (14.3)
% pupils eligible for free school meals^d^	20.1 (16)	11 (11.3)	16.8 (15.6)	12.9 (12.7)
School IMD score^e^	23.0 (13.9)	15.2 (10.2)	21.1 (12.5)	16.5 (11.0)

^a ^The number of pupils in Year 4 in 2005/2006. Information provided by Norfolk Local Education Authority

^b ^Number of schools answering 'yes' to the question Does your school have access to a specific hall for gym or sports (indoors)?

^c ^Playground area (area in which Year 5 children are allowed to play in on a fine day in the summer) indicated on a map by head teacher, and area calculated in a GIS

^d ^Free school meals are available to children from low-income families

^e ^Index of Multiple Deprivation score 2007 for the lower super output area the school falls within.

All six of our outcome measures were statistically significantly associated with rainfall in adjusted models (all p < 0.001). Relative to days with no rain, children spent 9.4 minutes (95%CI 7.6 to 11.2) fewer in MVPA on days in the top category for rainfall (>3.4mm), were sedentary for 13.6 minutes (95%CI 10.4 to 16.8) minutes more, and had an average 85.9 counts per minute (95%CI 69.4 to 102.3) fewer over the whole day. Over the lunchtime period children spent 2.2 minutes (95%CI 1.7 to 2.7) fewer in MVPA, sedentary time increased by 2.4 minutes (95%CI 1.5 to 3.2), and cpm were 100.6 counts (95%CI 77.8 to 123.3) lower.

### Moderation by school policy

**Figure 1:**
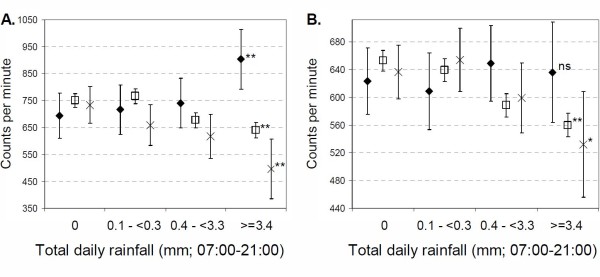
**Mean counts per minutes over (A) lunchtime (12:00-14:00), and (B) whole day (07:00-21:00) by categories of rainfall, at schools where: diamond = Children are kept indoors in wet weather, but indoor physical activities are allowed, square = Children kept indoors in wet weather, and indoor physical activities not allowed, cross = Children allowed outside in wet weather (no indoor physical activity options)**. Test for trend across rainfall categories; ns = not significant, * p < 0.05, ** p < 0.01. Error bars indicate 95% CI.

**Figure 2:**
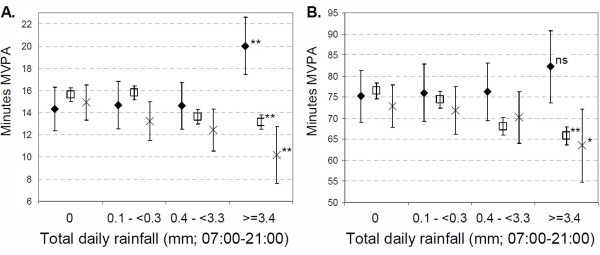
**Mean minutes spent in MVPA (A) lunchtime (12:00-14:00), and (B) whole day (07:00-21:00) by categories of rainfall, at schools where: diamond = Children are kept indoors in wet weather, but indoor physical activities are allowed, square = Children kept indoors in wet weather, and indoor physical activities not allowed, cross = Children allowed outside in wet weather (no indoor physical activity options)**. Test for trend across rainfall categories; ns = not significant, * p < 0.05, ** p < 0.01. Error bars indicate 95% CI.

Figure 1 shows average cpm by rain category and school policy at both the lunchtime (A) and whole day (B) periods. Over lunchtime, there was a decrease in overall activity over the categories of rainfall amongst those allowed outside (average cpm 238.1 counts (95%CI 108.2 to 368.0) lower on wettest days relative to dry days), and those kept in but not allowed to be active (average cpm 109.7cpm lower; 95%CI 71.37 to 147.9 on wettest days relative to dry days). On the wettest days those allowed out were significantly less active than either of the groups kept indoors; cpm were 145 counts (95%CI 31.6 to 259.6) lower than those not allowed to be active, and 408.0 counts (95%CI 250.9 to 565.1) lower than those who were allowed to be active. Among those allowed to be active indoors, cpm showed some increase with increasing rainfall, with average values 209.6 counts (95%CI 69.9 to 349.2) higher on the wettest days compared to days with no rain. For the whole day, cpm decreased with increasing rainfall among those not allowed to be active indoors and those allowed outside (although differences were not statistically significant among this group). A similar pattern was seen in minutes spent in MVPA (Figure 2), with those children who were allowed out generally exhibiting less MVPA with increasing rainfall. On the wettest days those allowed to be active indoors did 9.8 minutes (95%CI 6.2 to 13.5) more MVPA over lunchtime, and 18.7 (95%CI 6.6 to 30.8) minutes more over the whole day than those allowed outside.

**Figure 3:**
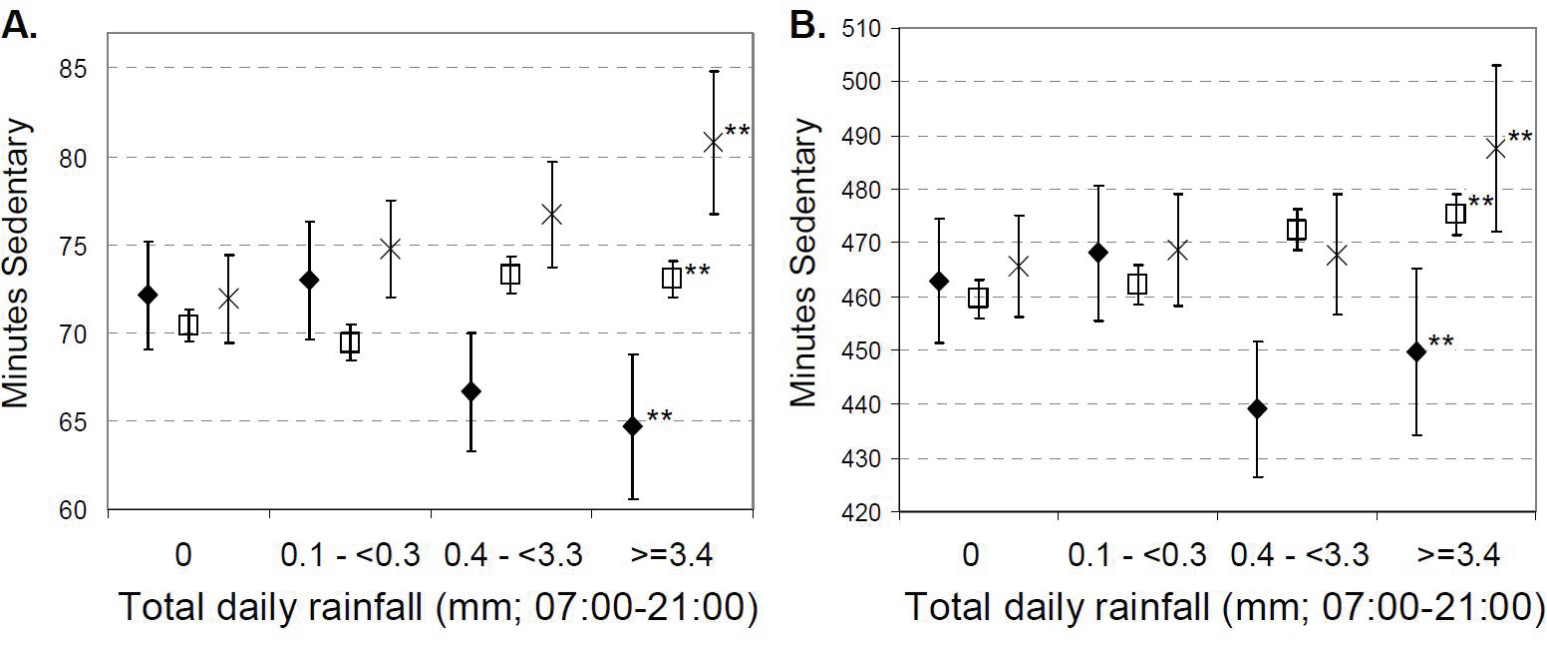
**Mean minutes spent sedentary over (A) lunchtime (12:00-14:00), and (B) whole day (07:00-21:00) by categories of rainfall, at schools where: diamond = Children are kept indoors in wet weather, but indoor physical activities are allowed, square = Children kept indoors in wet weather, and indoor physical activities not allowed, cross = Children allowed outside in wet weather (no indoor physical activity options)**. Test for trend across rainfall categories; ns = not significant, * p < 0.05, ** p < 0.01. Error bars indicate 95% CI.

When sedentary time was considered (Figure 3), the patterns were generally the opposite of those observed for cpm and MVPA. Over lunchtime, those allowed outside were increasingly more sedentary with increased rainfall, and on the wettest days spent 8.7 minutes (95%CI 4.1 to 13.6) more time sedentary than on dry days, and 16.1 minutes (95%CI 10.3 to 21.9) more than those allowed to be active indoors. For that group, sedentary time decreased with increasing rainfall, with 7.4 minutes (95%CI 2.3 to 12.6) less sedentary time on the wettest days relative to the driest. Those not allowed to be physically active indoors spent 2.7 minutes (95%CI 1.3 to 4.1) more time sedentary on the wettest days compared with the driest, but were still less sedentary on the wettest days compared to those allowed outside (mean difference 7.7 minutes, 95%CI 3.5 to 11.9). Looking at the whole day, similar patterns to those seen over the lunchtime period are evident.

## Discussion

We investigated the associations between rainfall and objectively measured physical activity in 9-10-yr old British children. We found that overall physical activity, and minutes spent in MVPA decreased with increasing rainfall, while sedentary time increased. However, school break time policies were seen to significantly modify these associations. Contrary to our expectations, being allowed outdoors at break time during wet weather was associated with a decrease in MVPA and an increase in sedentary time on the wettest days. It is possible that when they are outside in wet weather children focus on staying dry rather than playing, and therefore spend their break times standing under shelter. Others have found that children are more active when they are outdoors [22-24], but it appears that this association is dependant on rain conditions, and that children who are allowed outdoors in wet weather exhibit similar activity levels to those kept indoors in a classroom environment.

We found that it was the children who were given the opportunity to be physically active indoors in wet weather who were able to remain most active. Children in this group had significantly higher counts per minute, spent longer in MVPA and were less sedentary than those allowed outdoors, and those kept indoors and not given physical activity opportunities. These differences were most obvious over the lunchtime period, but were also statistically significant over the whole day, where the difference in MVPA on the wettest days between those allowed out and those allowed to be active indoors was over 18 minutes; equivalent to almost a third of a child's recommended daily total of MVPA.

Our findings suggest that a focus on encouraging indoor physical activities in wet weather may help children remain active during school hours, and may also contribute to improved daily physical activity levels on wet days. However, it is unclear what the long term effect of such a policy may be. In adults it has been observed that those who perceive the weather as a barrier are less likely to walk for exercise, and spend less time walking in their home neighbourhoods [25]. In the longer term, a policy of keeping children indoors in wet weather may enhance the perception that the weather is a barrier to physical activity, and this could be detrimental to their physical activity levels as they track into adulthood. It is also noteworthy that six of the seven (86%) schools that allowed indoor physical activities had separate indoor sports halls, compared with 60% of the schools that did not allow indoor PA. This may reflect the fact that schools are limited in their policy choices by their facilities, and where the space is not available for indoor physical activities in wet weather, schools may be advised to focus on encouraging greater activity outdoors, possibly by the provision of adequate wet weather clothing, or sheltered all-weather play spaces. Nevertheless, further work is required to examine how the associations observed here may vary as children age, and also what specific steps may be most effective to engage children in outdoor physical activity in wet weather.

This study has a number of strengths and weaknesses. In terms of strengths, we were able to recruit a large sample of schools and pupils, and objectively measured both physical activity and rainfall. This allowed us to examine in detail activity of differing intensities at specific points in the day, and gave variation in rainfall exposure within subjects. Furthermore our measures of school policy were reported directly by head-teachers rather being taken from secondary sources.

The study's weaknesses include the restricted period over which data were collected; our study was conducted over just one season (mid-April to mid-July), which saw unusual weather conditions across the UK [23]. A six-week dry episode starting in the second week of March included the warmest April on record, and preceded the wettest summer since 1912 [26]. The first three weeks of our study period saw no rainfall, while later months, especially June and July, were considerably wetter, giving a rainfall total for the period double the average [27]. We cannot say whether the associations we observed would usually be seen at this time of year, or at other times of the year, nor begin to disentangle seasonal and weather effects. For example, it may be that the magnitude of wet weather effects is greater in winter when temperatures are lower than during our sampling period. A further limitation is that our sample of children is from a restricted age range and we do not know if associations we observed would be apparent in younger or older groups.

Participants provided up to six days of activity data, but these were over consecutive days (or two separate sets of consecutive days, separated by a weekend), potentially limiting the variability of rainfall each participant was exposed to. While our rainfall data were from an official source, and were available at hourly intervals, they were only from two locations. The mean distance for schools to nearest weather station was 29km (SD:14km), and while we were able to derive daily rain totals with the reasonable confidence that they would be representative of the daily rainfall for each school, the distance between stations and schools meant that we did not attempt to measure whether it was actually raining during the lunchtime period. Nevertheless, we hypothesise that lunchtime bad weather policies at schools may be activated on days when there is rainfall generally observed or forecasted during the day, diminishing the importance of actual conditions during the lunch period.

While accelerometers provide an objective measure of physical activity, they are not without their limitations. They have a limited ability to assess activity while the wearer is cycling [28], and must be removed altogether during aquatic activities. However, these are unlikely to be activities undertaken by children during their lunch break so their under-measurement is not likely to have impacted our findings. A further limitation of hip worn accelerometers is that they do not capture upper body movements and hence we may have underestimated the activity levels of children who sit but are very active with their arms during periods of the day. As physical activity data were not available at a less than hourly temporal resolution, we used a broad definition of lunchtime (12noon - 2pm) which encompassed the lunch period at all schools. However, this period would also include non-break time, and any activity undertaken then.

The majority of the schools in our study had similar policies for break times in bad weather, namely children were kept indoors and not given the opportunity to be physically active. Despite the relatively small number in the other two policy groups, we were still able to detect significant differences in children's activity levels on wet days. Twenty-seven of our schools reported neither allowing physical activities nor additional sedentary opportunities indoors in bad weather. We do not know what children at these schools are allowed to do at break in bad weather, although their activity levels were not significantly different from those kept in and allowed to do additional sedentary activities (results not presented). The questions in the head teacher questionnaire were framed around 'bad' weather. In a UK summer context this would typically be synonymous with rainfall, but we did not ask schools to actually define what 'bad' weather meant to them. A final limitation is that schools in Norfolk, and consequently our sample, have a low proportion of non-white pupils, which potentially limits the generalisability of our findings to more ethnically diverse populations.

## Conclusions

We have found that increased rainfall was associated with a decrease in children's physical activity. However, schools' policies can modify this association, and providing indoor physical activities in wet weather may help children maintain physical activity levels. Further investigation is needed into the long term implications of such a policy, and into how these associations may vary by season.

## Abbreviations

CI: Confidence Interval; cpm: counts per minute; FMI: Fat Mass Index (Fat Mass(kg)/height(m)2); MVPA: Moderate-to-Vigorous Physical Activity (defined as >2000 counts per minute); PA: Physical Activity; SPEEDY: 'Sport, Physical activity and Eating behaviour: Environmental Determinants in Young people'; a cross-sectional study investigating individual and collective determinants of diet and physical activity in 9-10 year old children

## Competing interests

The authors declare that they have no competing interests.

## Authors' contributions

FH conceived the study and its design, performed the statistical analysis and drafted the manuscript. APJ and GB were involved with the study design. All authors were involved with data interpretation, critical revisions of the paper and provided approval for its publication.
